# Phase separation by the SARS-CoV-2 nucleocapsid protein: Consensus and open questions

**DOI:** 10.1016/j.jbc.2022.101677

**Published:** 2022-02-04

**Authors:** Sean M. Cascarina, Eric D. Ross

**Affiliations:** Department of Biochemistry and Molecular Biology, Colorado State University, Fort Collins, Colorado, USA

**Keywords:** SARS-CoV-2, phase separation, biomolecular condensate, membraneless organelle, intrinsically disordered region, nucleocapsid protein, innate immunity, stress granule, ERGIC, ER-Golgi intermediate compartment, gRNA, genomic RNA, IDR, intrinsically disordered region, MD, molecular dynamics, N, nucleocapsid, PS, phase separation, RNP, ribonucleoprotein

## Abstract

In response to the recent SARS-CoV-2 pandemic, a number of labs across the world have reallocated their time and resources to better our understanding of the virus. For some viruses, including SARS-CoV-2, viral proteins can undergo phase separation: a biophysical process often related to the partitioning of protein and RNA into membraneless organelles *in vivo*. In this review, we discuss emerging observations of phase separation by the SARS-CoV-2 nucleocapsid (N) protein—an essential viral protein required for viral replication—and the possible *in vivo* functions that have been proposed for N-protein phase separation, including viral replication, viral genomic RNA packaging, and modulation of host-cell response to infection. Additionally, since a relatively large number of studies examining SARS-CoV-2 N-protein phase separation have been published in a short span of time, we take advantage of this situation to compare results from similar experiments across studies. Our evaluation highlights potential strengths and pitfalls of drawing conclusions from a single set of experiments, as well as the value of publishing overlapping scientific observations performed simultaneously by multiple labs.

SARS-CoV-2, the virus responsible for COVID-19 and the ongoing pandemic, has exacted an enormous toll on human health, with >5.6 million deaths and >350 million infections currently attributed to the virus (according to the World Health Organization data, https://covid19.who.int/, accessed on 1/26/22). The pandemic had already led to an estimated $16 trillion in global economic costs by October 2020 (1) and disrupted nearly every economic sector, including science. Over the past year and a half, extraordinary progress has been made in improving our understanding of this novel virus. Emerging experimental results implicate the SARS-CoV-2 nucleocapsid (N) protein as a critical viral factor mediating viral replication, viral genomic RNA (gRNA) packaging, and modulation of host-cell response to infection. Intriguingly, the N protein has the ability to undergo phase separation (PS), which is now considered a pervasive phenomenon organizing a broad diversity of biological processes in cells. In this review, we discuss emerging models, experimental results, and possible *in vivo* functions related to PS by the SARS-CoV-2 N protein.

Additionally, given the remarkable number of related publications on this topic within the span of ∼ 1 year, we leverage this unusual situation to evaluate how overlapping work, performed and published in parallel by independent groups, may shape resulting conclusions. In practice, science is often performed sequentially: one published discovery typically precedes, informs, and directs subsequent experimentation. The incentive structure in science, which rewards novelty, promotes this sequential model, and disincentivizes studies focused on replication and validation. One limitation of this sequential model is that subtle differences in experimental design can sometimes have a significant impact on experimental results and thus influence the direction of subsequent experiments. However, the sequential publication model was punctuated by the SARS-CoV-2 pandemic. Many labs with historically little or no prior experience in virology applied their respective areas of expertise to questions related to SARS-CoV-2. This abrupt reallocation of resources to the same topic of study by many labs organically created a scientific question of its own: what happens when multiple labs perform and publish closely related experiments in parallel? Here, we compare a set of recent and related studies reporting PS by the SARS-CoV-2 N protein to examine this question.

## The role of the N protein in virion formation and gRNA packaging

The prototypical function for coronaviral N proteins is to condense and organize gRNA in nascent virions (2). Virion formation occurs *via* the accumulation of the SARS-CoV-2 structural proteins [the spike (S), envelope (E), membrane (M), and N proteins] and gRNA at the ER-Golgi intermediate compartment (ERGIC) membrane. Multiple studies suggest that a single strand of SARS-CoV-2 gRNA forms dense, locally ordered ribonucleoprotein (RNP) regions consisting predominantly of N protein associated with the gRNA strand (3, 4, 5). Locally ordered RNPs may be further organized into more complex arrangements *via* clustering of RNPs in particular stoichiometries and geometries (3, 4), although other evidence and prior models of the SARS-CoV N protein suggest a more linear, helical RNP arrangement (2, 5, 6). These RNPs preferentially accumulate on curved membranes (3), indicating either that RNP association aids in membrane curvature during nascent virion formation, or that curved membranes are the preferred recruitment surface for RNPs. The N protein also interacts with a luminal domain (*i.e.*, in the interior of virions) of the M protein, which was proposed as a possible mechanism for mediating recruitment of N-containing RNPs to the ERGIC membrane (7). Some evidence suggests that the N protein of both SARS-CoV and SARS-CoV-2 may also interact with the E protein (8, 9). While precise detail regarding the interactions and arrangements of individual molecules within intact virions is still forthcoming, the N protein clearly plays a central role in gRNA compaction and organization in SARS-CoV-2 virions.

## What is “phase separation”?

PS by a protein involves the formation of two distinct yet coexisting phases from a well-mixed protein solution: a dense phase of high protein concentration and a dilute phase of low protein concentration (10). Subsequent to initial PS, the dense phase may also undergo additional phase transitions that change its material properties (11, 12). Consequently, condensates can exhibit material properties consistent with liquids, gels, or solids (13), and these properties can be influenced by many factors including protein sequence, protein concentration, the presence and concentrations of other molecules, and physical and chemical environment. One of the key features of proteins associated with PS is “multivalency,” which describes proteins with multiple binding sites for partner molecules. PS can occur in either single-component or multicomponent systems (14, 15). In a single-component system, PS is driven by homotypic interactions (*i.e.*, between two identical biopolymers), whereas co-PS in a multicomponent system is driven by heterotypic interactions (*i.e.*, between different biopolymers) or a combination of homotypic and heterotypic interactions. While no single type of domain is required in multivalent proteins to observe PS, certain domains appear to be more common among proteins known to phase separate. For example, a number of phase separating proteins contain RNA-binding domains, intrinsically disordered regions (IDRs), oligomerization domains, and low-complexity domains.

PS has gained recent attention in biology due to its connection with “biomolecular condensates” (10, 13, 16), which are membraneless organelles that are typically enriched in certain proteins and nucleic acids. Much like the dense phase observed *in vitro*, biomolecular condensates consist of a network of interactions between multivalent proteins and partner molecules (often nucleic acids, proteins, or other biopolymers). Many types of biomolecular condensates have been described, including (but not limited to) stress granules, P-bodies, nucleoli, nuclear speckles, germ granules, and Cajal bodies (10). Each type of biomolecular condensate is associated with distinct sets of constituent molecules, material properties, biological functions, stability, and regulation. Regardless of these differences, biomolecular condensation represents an elegant biological solution for organizing and concentrating groups of molecules in a regulatable and sensitive fashion.

## PS and the SARS-CoV-2 N protein

Given the prevalence, diversity, and importance of biomolecular condensates in eukaryotes, it is perhaps no surprise that some viruses are able to interact with and manipulate endogenous condensates or trigger the formation of entirely new viral condensates in host cells (17, 18). Shortly after the emergence of SARS-CoV-2, we proposed that the SARS-CoV-2 N protein would undergo PS *in vitro*, and that similar biophysical behavior *in vivo* might mediate the formation of RNA–protein condensates during viral RNA packaging into new virions, or modulate host-cell condensates (namely, stress granules) *via* direct physical interaction (19). In the ensuing months, many studies examining various aspects of the PS behavior of the SARS-CoV-2 N protein, including its role in viral RNA packaging, stress granule modulation, regulation of host-cell innate immune pathways, and regulation by host-cell kinases, were formally published (7, 20, 21, 22, 23, 24, 25, 26, 27, 28, 29, 30, 31, 32, 33, 34, 35, 36).

**Figure 1:**
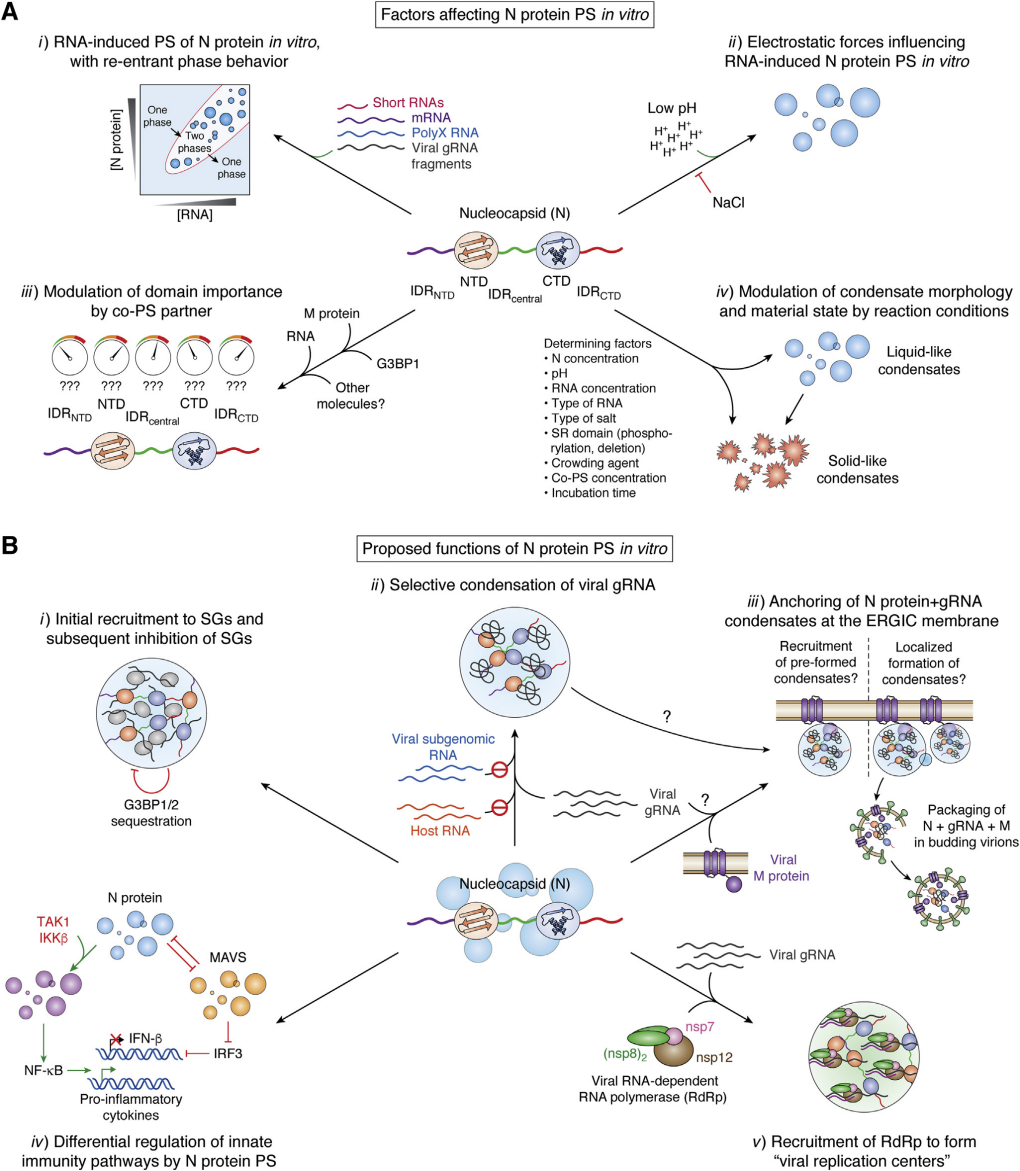
**N-protein PS regulation and function.***A*, N-protein PS *in vitro* is influenced by: i) re-entrant PS behavior with RNA of various lengths and sequences, ii) electrostatic forces, iii) interactions between specific N-protein domains and co-PS partners, and iv) experimental conditions or N-protein modifications that alter condensate morphology and material state. *B*, proposed *in vivo* functions associated with N-protein PS include: i) recruitment of N protein to stress granules (SGs), resulting in sequestration of key SG factors and subsequent inhibition of SGs; ii) selective condensation of SARS-CoV-2 gRNA by co-PS with the N protein, iii) anchoring of N+gRNA condensates at the ERGIC membrane by co-PS with the SARS-CoV-2 M protein during the formation of new virions; iv) regulation of host-cell innate immune pathways, including upregulation of cytokine production *via* co-PS with NF-κB activators, as well as inhibition of IFN-β expression *via* suppression of host-cell MAVS PS; and v) recruitment of SARS-CoV-2 RNA-dependent RNA polymerase (RdRp) to form “viral replication centers.” PS, phase separation.

Figure 1 highlights the factors affecting N-protein PS *in vitro* and the proposed functions of N-protein PS *in vivo*, each of which is discussed in the ensuing sections. Additionally, we compare the N-protein domains purported to be critical for PS and, more broadly, what can be learned from a “consensus” view resulting from many related studies published in a short timeframe. We would like to note that while we have done our best to faithfully interpret the available data, not all studies present rigorous quantification of PS and quantification methods differed between studies; therefore, our conclusions are based at least to some degree on our subjective interpretation.

## *In vitro* PS by the SARS-CoV-2 N protein is induced by RNA, predominantly driven by electrostatic interactions, and regulated by phosphorylation of the SR domain

PS has often been associated with RNA-binding proteins containing prion-like or other low-complexity domains (37, 38, 39). RNA itself is capable of undergoing PS (40), can often induce PS of specific proteins at lower protein concentrations (41), and can regulate the material properties of condensates in a variety of ways [reviewed in (42)]. The SARS-CoV-2 N protein contains two structured domains capable of binding RNA (43, 44, 45, 46, 47), as well as multiple flanking IDRs that enhance RNA binding (44, 47). While N protein alone typically exhibited weak or undetectable PS in the majority of studies (7, 20, 21, 27, 29, 30, 31, 32, 33), RNA almost universally induced PS of the SARS-CoV-2 N protein across all studies evaluated in depth (Fig. 1*A*i). RNAs of varying lengths and sequences can induce N-protein PS to varying degrees, suggesting that this process is somewhat nonspecific *in vitro* (though the formation of N+RNA condensates *in vivo* may exhibit greater sequence specificity, as discussed in a later section). For studies that tested a wide range of RNA concentrations, exceedingly high amounts of RNA tended to inhibit PS (7, 22, 28, 32), which is consistent with re-entrant phase behavior due to an imbalance in the stoichiometries of constituent molecules.

Electrostatic forces were consistently implicated across studies in mediating or regulating RNA-dependent PS (Fig. 1*A*ii). PS of proteins is often sensitive to salt concentrations and types of salts used (48), which is generally presumed to reflect electrostatic driving forces for PS. Lower salt concentrations were typically associated with enhanced N-protein PS (20, 25, 28, 29, 32, 33, 35, 36), suggesting that electrostatic interactions play an important role in PS. Additionally, in the few studies that examined N-protein PS across a range of pH conditions, lower pH was associated with enhanced PS (20, 32).

Phosphorylation of serine residues within the S/R-rich low-complexity domain (“SR domain”) consistently led to less viscous and more dynamic liquid-like droplets (29, 30), presumably by reducing N-protein affinity for RNA and increasing intramolecular and intermolecular N-N interactions (29). Additionally, S→D phosphomimetic substitutions within the SR domain also decreased viscosity and correspondingly increased dynamic exchange of the N protein within condensates, whereas nonphosphorylatable S→A substitutions or SR domain deletion led to more solid-like assemblies with slower recovery rates (7).

## PS involves a multivalent N-protein architecture, but little consensus is reached on which domains are essential

PS involves multivalent interactions between condensate constituents, resulting in a meshwork of interactions that are fundamental for condensate formation and maintenance (10). The SARS-CoV-2 N protein contains at least five domains, all of which have characteristics that are commonly associated with PS behavior: three IDRs (IDR_NTD_, IDR_central_, and IDR_CTD_) and two structured domains typically referred to as the RNA-binding domain (NTD) and the dimerization domain (CTD). However, neither the RNA binding nor the oligomerization activities are limited to the NTD or CTD (43, 44, 45, 46, 47, 49), respectively, further highlighting the complex multivalent nature of the N protein.

Examination of all possible combinatorial deletions of N-protein domains is experimentally feasible (*n* = 30) but has not been reported in a single study: all studies to date tested only a subset of domain deletions. Despite substantial overlap in the domain deletions or mutations tested across studies, many groups implicate distinct (and sometimes conflicting) regions as critical for N-protein PS (Fig. 1*A*iii). For example, Lu *et al.* (7) identified multiple constructs lacking either the NTD or CTD that were still capable of undergoing PS and implicate the IDR_central_ as critical for PS, while Zhao *et al.* (20) observed complete inhibition of PS upon deletion of the NTD or CTD. In yet another study (32), deletion of the IDR_central_ completely abolished PS, and deletion of the IDR_NTD_ and structured CTD nearly completely eliminated PS. Meanwhile, two other studies implicate the IDR_NTD_ as critical for co-PS with the canonical stress granule marker, G3BP1 (31), and recruitment to G3BP1-positive stress granules (31, 33).

In order to compare the PS behavior of N-protein domain deletions or amino acid substitutions across studies, we devised a qualitative six-point scale to categorize the relative effect of each modification on N-protein PS. For each study, constructs were compared with the wild-type control from that study, under the same set of conditions. Each deletion or substitution construct was categorized as exhibiting a large increase in PS (+2), a small increase in PS (+1), no change in PS (0), a small decrease in PS (-1), a large decrease in PS (-2), or complete abolishment of PS (-3). All categorized constructs across 12 major studies were combined and sorted based on qualitative score. This scoring is clearly imperfect due to the challenges of comparing across different studies, which utilize different quantification methods; thus, these values are solely intended to indicate the directionality and approximate strength of changes in PS and should not be interpreted as rigorous quantification.

Purified N protein by itself is capable of undergoing PS under particular experimental conditions [particularly low NaCl or KCl concentrations, high ZnCl_2_ concentrations, or high temperature; (21, 25, 28, 35, 36)]; however, the majority of studies report little or no PS by the N protein in the absence of RNA under the conditions tested (7, 20, 21, 27, 29, 30, 31, 32, 33). Consequently, most experiments probing the effects of N-protein domain deletion or mutation on N-protein PS were performed in the presence of RNA, where N-protein PS was robust and within a range suitable for detecting both increases and decreases in PS when mutating the N protein.

**Figure 2:**
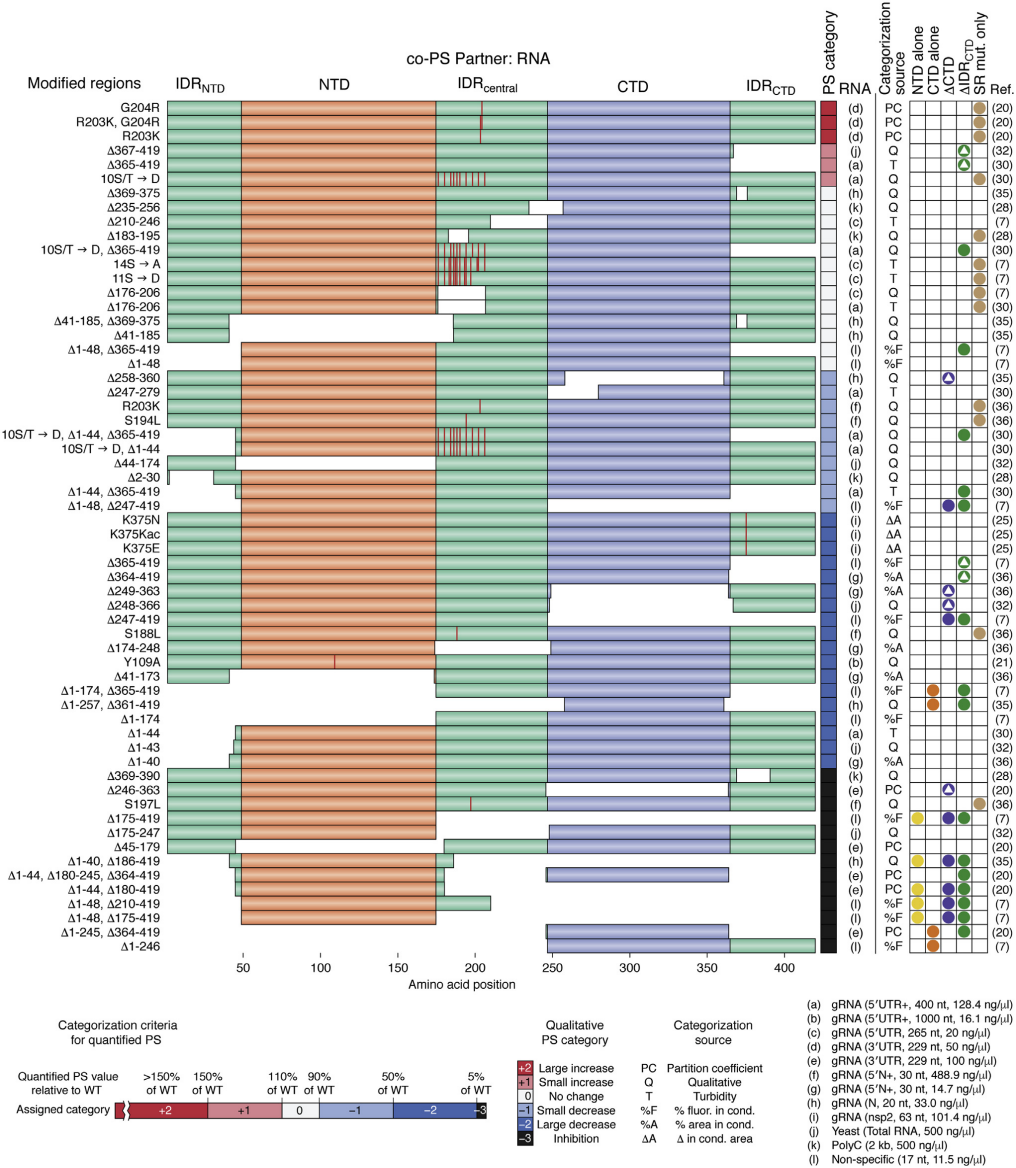
**Qualitative assessment of the effects of N-protein deletion and substitution mutants on co-PS with RNA.** N-protein mutants were categorized on a six-point qualitative PS scale (“PS Category” from −3 to 2, 0 included) to reflect the degree of PS relative to wild-type N protein within each study. Whenever available, quantification of PS (rather than representative images) was used for our categorization (see scale, *bottom*). Quantification methods included N-protein partition coefficient (20), area occupied by condensates (25, 36), turbidity (7, 30, 32), and percentage of RNA fluorescence partitioning to condensates (7). When only representative microscopy images were available, we subjectively estimated relative changes in PS by considering the apparent total area of the image corresponding to condensates. Deleted regions are indicated by gaps in the colored bars. Locations of substitutions are indicated in red. Constructs are grouped based on PS category but are not meaningfully rank ordered within each PS category. “NTD alone” (*yellow dots*) and “CTD alone” (*orange dots*) are defined as constructs consisting only of NTD or CTD with no more than one other IDR. “ΔCTD” (*purple dots*) and “ΔIDR_CTD_” (*green dots*) indicate any construct in which deletion of the CTD or IDR_CTD_ was performed, either alone (*dots* with *embedded triangles*) or in combination with additional mutations (*dots* without *embedded triangles*). “SR mut. only” (*tan dots*) indicates constructs in which the SR domain (which is located within the IDR_central_) is mutated, with all other regions remaining unchanged. RNA descriptions include the type, source, length, and concentration, respectively. RNA concentrations were converted from units of μM to ng/μl when necessary using specified RNA sequences in the original study. The origin of the 17-mer “nonspecific” RNA (AAGCAGCUAAGAGCGAA) from Lu *et al.* (7) was not explicitly described. A “+” next to the RNA source indicates that a portion of the sequence is also derived from neighboring regions within the RNA of origin. IDR, intrinsically disordered region; PS, phase separation.

Importantly, each study used different experimental conditions, which may impact the experimental results. For example, the types of RNA (*e.g.*, total yeast RNA, homopolymeric RNA, and various SARS-CoV-2 gRNA fragments), lengths of RNA, and concentrations of RNA likely all influence N protein PS, yet these features differed substantially between studies (Fig. 2). Additional differences in reaction conditions such as salt concentration, pH, temperature, and protein concentration might also affect the N-protein domains involved in co-PS. While these differences make exact comparisons between studies difficult, they also offer an opportunity: multiple studies can help reveal which domains are robustly involved in co-PS with RNA across experimental conditions and which are specific to a subset of the tested reaction conditions.

**Figure 3:**
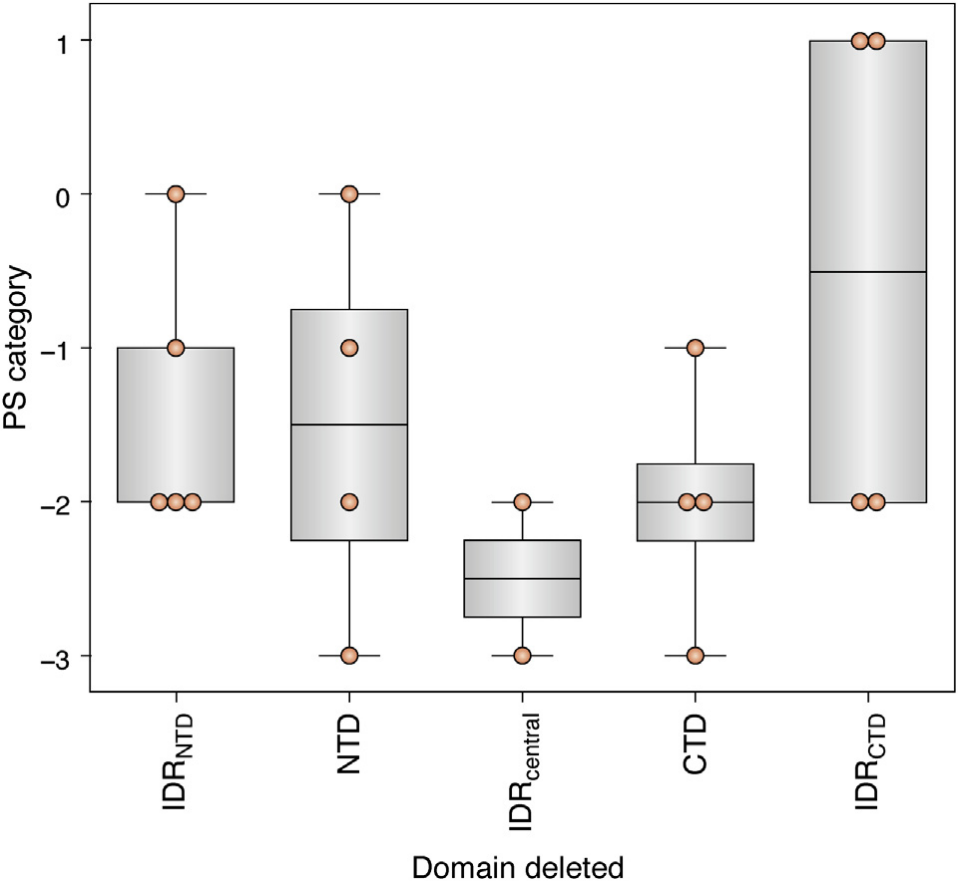
**PS categories for each single-domain deletion within the N protein.** PS categories for N-protein variants consisting of only a single-domain deletion were grouped and plotted. Since putative N-protein domain boundaries varied between studies, single-domain deletions were defined as N-protein variants for which ≥60% of one domain was deleted, with no other region mutated. PS, phase separation.

While no universal domain contributions to co-PS with RNA are immediately apparent, a number of trends are discernible. First, both the NTD alone and the CTD alone show weak or undetectable co-PS with RNA (Fig. 2, yellow dots and orange dots, respectively), even though they are typically considered the canonical RNA-binding and dimerization domains, respectively. This is consistent with a multivalent architecture required for PS. By extension, deletion of larger total segments was more likely to be associated with greater inhibition of PS. Second, the CTD was consistently required to observe co-PS with RNA at a level equivalent to or better than wild-type N protein: of the 19 proteins with a qualitative score ≥0, none had a complete CTD deletion (Fig. 2, purple dots, and Fig. 3). In contrast, two of the 19 proteins contained an NTD deletion, two of the 19 contained an IDR_NTD_ deletion, and four of the 19 contained a complete IDR_CTD_ deletion (Fig. 2, green dots and Fig. 3). Third, while two point mutations in the SR domain (which resides in the IDR_central_) were reported to have a strong negative effect on co-PS with RNA, most mutations or deletions had no more than a mild effect on co-PS with RNA, and many even enhanced PS compared with wild-type N protein (Fig. 2, tan dots). This is consistent with the observations noted above [and somewhat contrary to our *a priori* expectations (19)] that the SR domain tends to be a modulator rather than a driver of *in vitro* PS and is most likely governed by host-cell kinases (7, 29, 30, 50, 51). However, complete deletion of the full IDR_central_ strongly reduced or completely inhibited condensate formation (Fig. 3), suggesting that a larger region of the IDR_central_ plays a more deterministic role in condensate formation.

**Figure 4:**
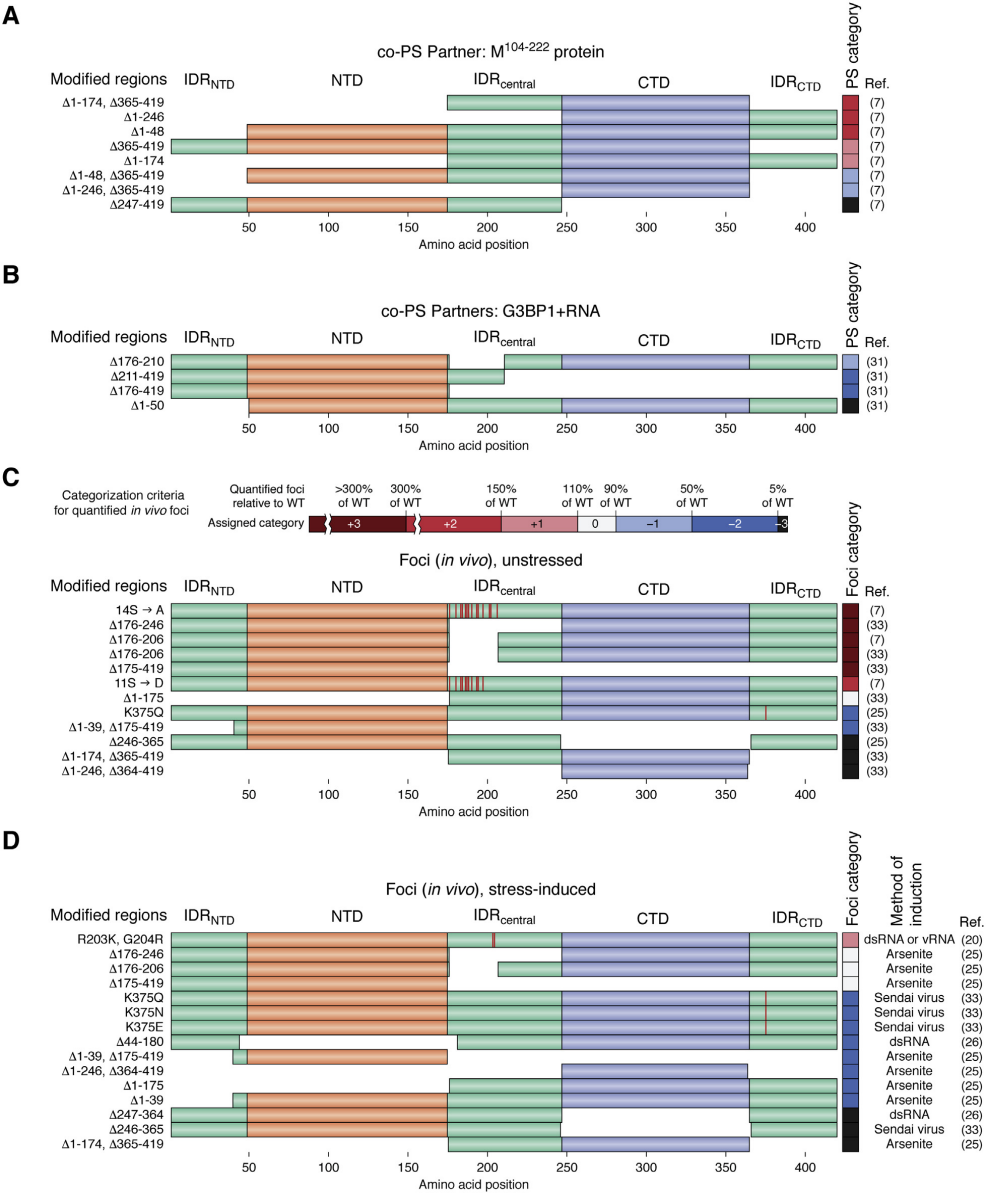
**Qualitative assessment of the effects of N-protein deletion and substitution mutants on co-PS with non-RNA molecular partners and *in vivo* foci.** N-protein mutants were categorized on the same scale as indicated for Figure 2, but with the non-RNA co-PS partners M^104–222^ protein (*A*), G3BP1+RNA (*B*), or the formation of foci *in vivo* (*C* and *D*). Foci formed in the absence (*C*) or presence (*D*) of stress were scored separately, and the method of stress used to induce foci is indicated at the *top* of panel *C*. Since multiple groups observed a low percentage of cells with foci in the absence of stress, we added a new category (+3) to further resolve large increases in the formation of foci relative to WT. Additionally, since regulation of stress granules is only one of many possible *in vivo* functions proposed for N-protein PS, colocalization with stress granules was not a requirement for consideration in panels *C* and *D*. The study by Luo *et al.* (31) was not included in panels *C* and *D* because representative images often showed rare instances of foci formation and were not intended to reflect the population-wide degree of foci formation. PS, phase separation.

Other studies examined N-protein mutants in the context of co-PS with a soluble fragment (residues 104–222) of the SARS-CoV-2 M protein (7), co-PS with G3BP1+RNA (31), or the ability to form foci *in vivo* (33) (Fig. 4). These different contexts implicate different regions of the N protein as important or essential for PS behavior. For example, with RNA as the partner molecule, deletion of the IDR_NTD_ resulted in no apparent change in co-PS, a slight decrease in co-PS, or a severe decrease in co-PS, depending on the study (Figs. 2 and 3). However, IDR_NTD_ deletion substantially increased co-PS with the M^104–222^ protein (Fig. 4*A*), yet completely abolished co-PS with G3BP1+RNA (Fig. 4*B*), neither of which were observed in any of the studies examining co-PS with RNA alone. Furthermore, deletion of the NTD and IDR_NTD_ individually resulted in a slight reduction and severe reduction, respectively, in stress-induced foci formed *in vivo*, and deletion of the two domains together completely abolished stress-induced foci (Fig. 4*D*). Similarly, a mutant N protein consisting of only the IDR_central_ and the CTD strongly enhanced co-PS with the M^104–222^ protein (Fig. 4*B*) but strongly inhibited co-PS with RNA (Fig. 2) and completely abolished the formation of foci under both stress and nonstress conditions *in vivo* (Fig. 4, *C* and *D*).

Finally, consistent with *in vitro* results suggesting that the SR domain acts as a modulator of phase behavior, substitutions within the SR domain or deletion of the SR domain all tended to increase the formation of N-protein foci in the absence of stress (Fig. 4*C*) and formed similar levels of stress-induced foci (Fig. 4*D*), further supporting a regulatory role of the SR domain in the formation of (or recruitment to) biomolecular condensates *in vivo*. However, the properties of foci formed by SR domain mutants often varied based on the specific mutation. For example, S→D phosphomimetic substitution resulted in a slight increase in the number of cells with foci compared with wild-type but a similar FRAP recovery rate, whereas nonphosphorylatable S→A substitution or complete deletion of the SR domain greatly increased the number of cells with foci relative to wild-type N protein but exhibited slow FRAP recovery rates (7). Interestingly, upon deletion of the IDR_central_, the N protein robustly formed foci even in the absence of stress *in vivo* (as described above), yet similar deletions strongly reduced or eliminated N+RNA co-PS *in vitro* (Fig. 2), illustrating one example in which the importance of a domain may differ between a simplified *in vitro* setting and an *in vivo* setting.

Collectively, these results suggest that the domains required for N-protein co-PS depend on available co-phase-separating molecules and experimental conditions, and may differ dramatically *in vivo*. Different co-PS partners can, in some cases, lead to differences in the N-protein domains important for co-PS. Similarly, even for a single type of co-PS partner (*e.g.*, RNA), differences in reaction conditions might also affect the N-protein domains involved in co-PS. While some degree of condition dependence is to be expected, this highlights a key caveat that must be considered when interpreting PS studies in general: if small changes in the experimental conditions can fundamentally change the role of different domains in promoting/inhibiting PS, then caution must be exercised when interpreting any single *in vitro* PS study. Multiple overlapping studies aid in delineating the domains that are robustly involved in co-PS across many conditions and those that play condition-sensitive roles in co-PS.

## Proteolysis, mutation, and posttranslational modification can influence N protein PS behavior *in vitro* and *in vivo*

**Figure 5:**
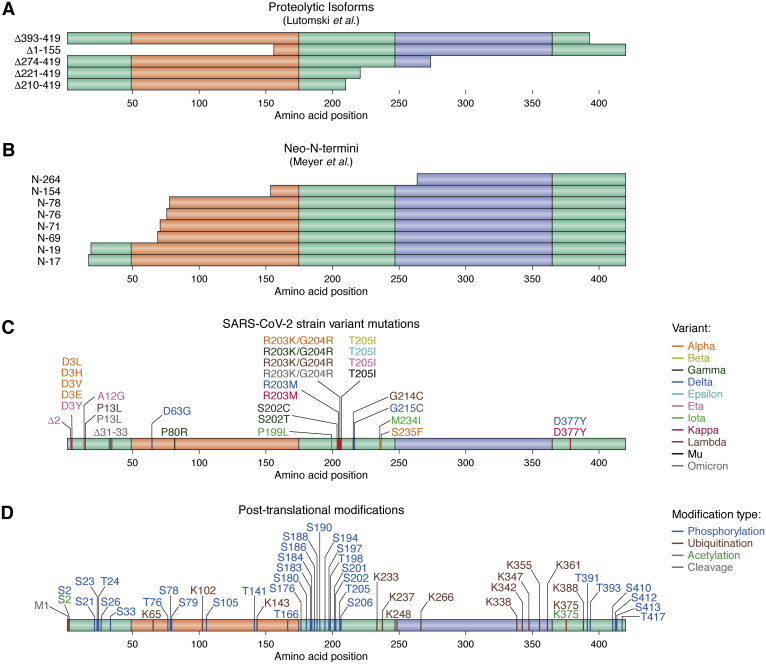
**Naturally occurring sequence variation within the N protein.** Proteolytic cleavage of the N protein results in a variety of stable isoforms when expressed in *E. coli* (*A*) and neo-N-termini when expressed in mammalian cells (*B*). Only C-terminal fragments are depicted in (*B*) since the employed experimental technique only detected new N-termini resulting from proteolysis (53), though it is possible that proteolytic events also generate stable N-terminal fragments. The locations of SARS-CoV-2 strain-specific mutations are depicted in (*C*) and were combined from the UCSC genome browser [https://genome.ucsc.edu/covid19.html; (90)] and the CoVariants website https://covariants.org/, both of which display data derived from the GISAID database [https://www.gisaid.org/; (91)]. The locations of N protein PTMs (*D*) were derived from (25, 28, 29, 92, 93, 94, 95). PTM, posttranslational modification.

Although domain deletions are often viewed simply as a molecular biology tool to ascertain which protein regions are important, two recent studies reported that the N protein is cleaved at multiple sites when expressed in bacterial or mammalian cells (52, 53) in a protease-dependent manner (53), generating a number of truncated protein isoforms that are remarkably similar to some of the domain deletion constructs previously tested for PS activity. Specifically, N_1–209_, N_1–273_, and N_156–419_ generated upon expression in *E. coli* (52) roughly correspond to variants consisting of IDR_NTD_+NTD+SR domain, IDR_NTD_+NTD + IDR_central_, and IDR_central_+CTD + IDR_CTD_, respectively (Fig. 5*A*). Similarly, N-terminal truncations at sites 170, 210, 263, and 373 when expressed in mammalian cells (53) roughly correspond to variants containing IDR_central_+CTD + IDR_CTD_, IDR_central_ (without the SR domain)+CTD + IDR_CTD_, CTD + IDR_CTD_, and IDR_CTD_ alone, respectively (Fig. 5*B*), with the potential for additional derivatives if the N-terminal truncations are cleaved at additional sites. The oligomerization state and RNA-binding capacity varied between N-protein isoforms (52), and the oligomerization state of specific isoforms was sensitive to pH. While oligomerization state may be related to PS, the PS activity of these variants *in vitro* or *in vivo* was not directly examined. Since the traditional method of examining protein localization and biomolecular condensation *in vivo* (*i.e.*, appending a fluorescent tag to the N- or C-terminus of a protein) cannot distinguish between stable proteolytic isoforms, this raises the possibility that specific proteolytic isoforms are involved in the formation of or recruitment to biomolecular condensates. Therefore, future studies (*e.g.*, expressing N-protein isoforms with two distinct fluorescent markers at the N- and C-termini) may be required to define potential isoform specificity for the N protein in biomolecular condensates *in vitro* and *in vivo*.

Alternative forms of N protein sequence variation that are regularly encountered *in vivo* and may affect N protein PS include naturally occurring mutations, particularly among well-defined SARS-CoV-2 strain variants (Fig. 5*C*), as well as posttranslational modifications (PTMs; Fig. 5*D*). Importantly, only two of the common variant mutations (R203K and G204R) within the N protein have been tested for their effects on N protein PS (Figs. 2 and 5*C*). The R203K/G204R double mutant directly enhances viral infectivity and fitness (54). Both mutations, either individually or in combination, strongly enhanced N+RNA co-PS, and the double mutant increased the formation of stress-induced foci *in vivo* in one study (20), although another group only tested the R203K mutant and observed a slight decrease in N+RNA co-PS (36). However, in the context of SARS-CoV-2 variants, these mutations always co-occur with additional, strain-specific mutations within the N protein. Similarly, while a number of studies have observed effects of PTMs on N protein PS, these studies have focused on phosphorylation within the SR domain and acetylation of K375, yet many more PTMs are distributed across the N protein sequence (Fig. 5*D*), including PTM sites that are identical or adjacent to mutated sites in SARS-CoV-2 strain variants (*e.g.*, S2, S33, S202, and T205), PTMs that occur within transient helices formed by the IDRs (22), and PTMs that occur on N protein residues that exhibit a selective increase in intramolecular or intermolecular interaction upon N protein PS (7, 28). Therefore, additional studies are required to fully elucidate the effects of proteolytic processing, strain-specific mutations, and PTMs on N protein PS *in vitro* and *in vivo*.

## Mechanistic studies implicate multiple, specific sites as potential mediators of N-protein homotypic and heterotypic interactions during PS

### Potential N-N homotypic interaction sites

**Figure 6:**
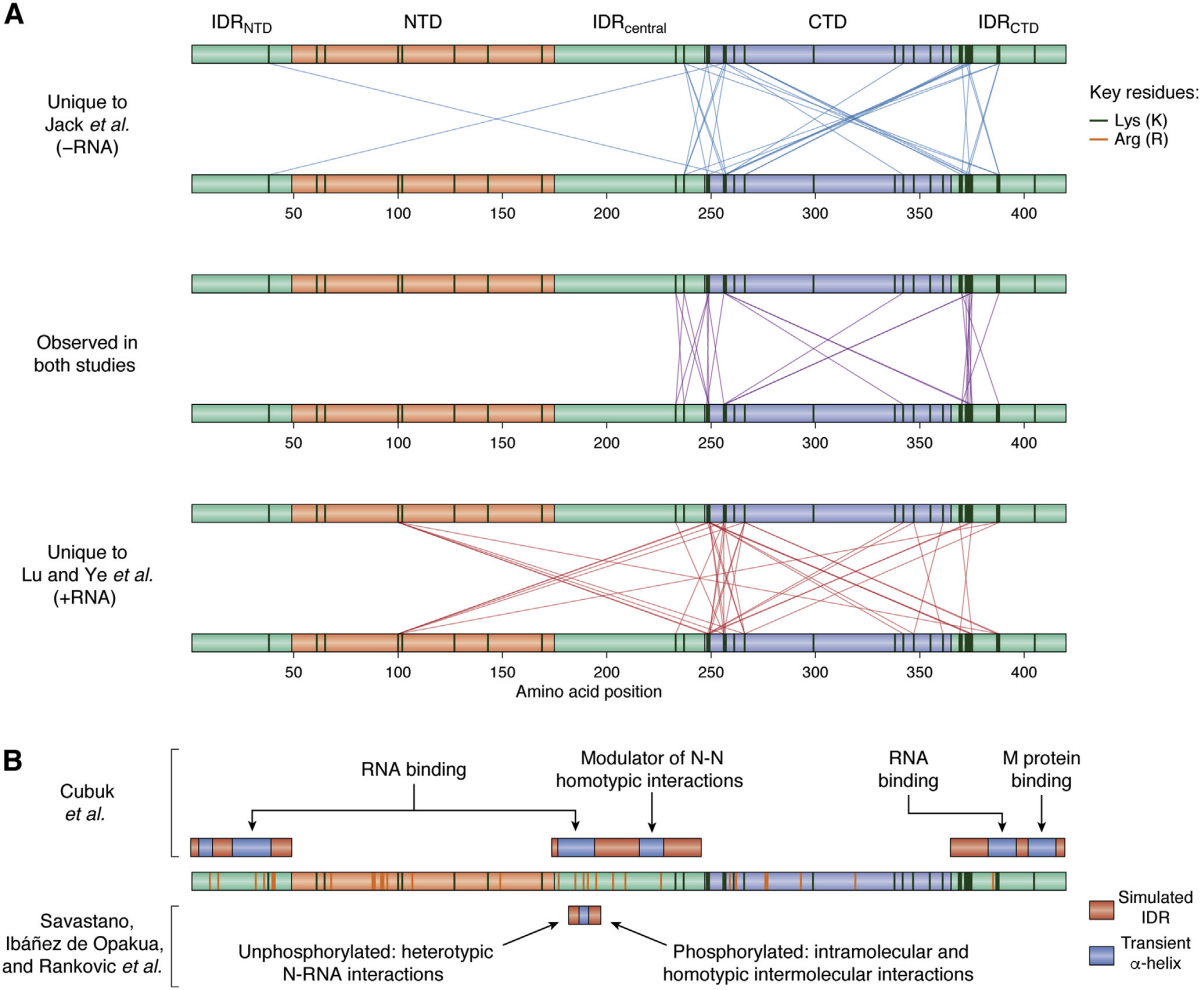
**Potential regions mediating N-protein PS implicated by cross-linking MS or MD simulations.***A*, N-protein sites with enriched K-K crosslinking upon PS identified in Jack *et al.* (28) (*top*), Lu *et al.* (7) (*bottom*), or both (*middle*). All K residues are indicated with *dark-green vertical lines* in the domain schematic (R residues are omitted from panel *A* for simplicity). *B*, IDRs studied by experimental and MD simulations, with those examined by Cubuk *et al.* (22) indicated above the N-protein schematic and the one examined by Savastano *et al.* (29) indicated below the N-protein schematic. *Red regions* indicate the IDRs used in MD simulations. *Blue subregions* indicate transient helices identified by MD simulation and/or experimental characterization within the IDRs. *Dark-green* and *orange vertical lines* indicate all K and R residues, respectively. Associated text descriptions indicate proposed functions for transient helices and IDRs. IDR, intrinsically disordered region; MD, molecular dynamics.

Domain deletion is an informative but low-resolution tool used to infer the regions of a protein important for activity and might fundamentally alter the landscape of interactions mediating N-protein PS. To gain additional mechanistic insight, multiple studies adopted complementary approaches to examine key residues mediating N-protein PS. Proximity-dependent cross-linking of lysine (K) residues within the N protein, followed by mass spectrometry (MS), implicated remarkably similar regions with significantly enriched interactions during N-protein PS (relative to the soluble state) in the presence of RNA (7) or in the absence of RNA (28). In both studies, K-rich regions immediately flanking the CTD (or partially within the CTD) interacted significantly more in the PS state than in the soluble state (Fig. 6*A*). A substantial number of identical interaction sites are enriched in both studies, and additional interactions observed in either study individually cluster near the same regions, suggesting that these observed interactions are robust.

The agreement between studies is somewhat surprising, given the difference in reaction conditions and the known importance of RNA in enhancing N-protein PS. Although N protein is capable of undergoing PS by itself in low-salt conditions [as used in (28)], PS by N protein alone is limited to homotypic (N-N) interactions, whereas co-PS with RNA involves both homotypic (N-N) and heterotypic (N-RNA) interactions. It is possible that similar homotypic interaction sites mediate N-protein PS regardless of the presence of RNA (7). However, these data should also be considered in light of technical limitations of the assay and in the context of the domain deletions results (Fig. 2). Except in rare cases, enriched interaction sites cannot be definitively classified as intermolecular N-N interactions (as opposed to intramolecular interactions that are selectively enriched in condensates), so it is unclear which of the sites are specifically mediating homotypic interactions during PS. Additionally, the bifunctional cross-linker used in both studies specifically mediates K-K cross-linking, which necessarily biases the results toward K-rich regions. Relatively few K residues are present outside of these regions and, of those residues, nearly all are found in the NTD (Fig. 6*A*), which may be occluded by RNA binding during co-PS with RNA. It is possible that additional homotypic interaction sites are enriched during PS but are undetectable in these cross-linking experiments. Finally, although these sites are enriched upon N-protein PS, their importance in mediating N-protein PS is not entirely clear. In a separate study (25), mutation or acetylation of K375—a site with significant cross-link enrichment upon PS in both cross-linking experiments (7, 28)—strongly attenuated N+RNA co-PS (Fig. 2). However, deletion of residues 369 to 375 had no apparent effect on N+RNA co-PS, deletion of much larger region (residues 369–390) completely abolished N+RNA co-PS, and deletion of the entire IDR_CTD_ had mixed effects across studies, sometimes even enhancing N+RNA co-PS (Fig. 2). Therefore, while the PS-dependent cross-linked sites appear to be robust, additional studies may be required to improve our mechanistic understanding of homotypic N-protein interaction during PS.

### Potential heterotypic N-protein interaction sites with co-PS partner molecules

Aside from the well-established surfaces for RNA binding in the structured NTD and CTD (45), all three IDRs of the N protein are known (44, 47) or expected (55) to contribute to RNA binding. Molecular dynamics (MD) simulations and accompanying NMR experiments involving a segment of the SR domain (amino acids 182–197) identified a very short, transient α-helix that mediated interaction between the SR domain and RNA (Fig. 6*B*) (29). R189, one of the key residues within the transient helix, exhibits an unusually low mutation frequency for this region, suggesting an important role for this position in SARS-CoV-2 viability. Indeed, in a separate study, a single substitution of an immediately adjacent residue (S188L) strongly attenuated N+RNA co-PS, and two more-distant substitutions within this region (S194L and S197L) slightly reduced or completely abolished N+RNA co-PS, respectively (Fig. 2). Furthermore, phosphorylation of even a single S within residues 182 to 197 decreased N-RNA heterotypic interactions and increased intramolecular and homotypic intermolecular interactions *via* interaction between the added phosphate and arginine residues within the SR domain (29), providing a potential explanation for the modulation of N+RNA condensates by SR domain phosphorylation.

A complementary set of MD simulations revealed multiple transient α-helices (H2, H3, and H5) within all three IDRs (Fig. 6*B*) that are proposed to aid in N-RNA interaction *via* orientation of arginine residues along the same face of the α-helix (22). Additionally, one segment in the IDR_CTD_ formed a transient amphipathic α-helix proposed to mediate N-M interaction, though N-protein co-PS with the M protein was slightly enhanced by deletion of IDR_CTD_ in a separate study (Fig. 4*A*). In many cases, the probability of α-helix formation within two of the IDRs was affected by neighboring structured regions, either by attractive interactions (*e.g.*, between the CTD and helix H6 in IDR_CTD_) or by repulsive interactions (*e.g.*, between the NTD and helix H3 in the SR domain). This raises the intriguing possibility that IDR conformations may be regulated by the availability of the structured domains. For example, interaction between the CTD and IDR_CTD_ may serve a weak autoinhibitory role that is disrupted by a change in environmental conditions, CTD dimerization, or interaction with RNA, thereby freeing the IDR_CTD_ and favoring α-helix formation by this region.

In summary, cross-linking/MS experiments and MD simulations have provided mechanistic details of the homotypic and heterotypic interaction sites that may mediate N-protein PS, though further studies are likely required to fully reconcile these observations with apparently contradictory effects of domain deletions on N-protein PS.

## N-protein condensate size, morphology, and material state vary substantially between studies and are highly condition-sensitive

The term “phase separation” has been used to describe the formation of condensates with a wide range of densities, sizes, morphologies, and material properties. For the N protein, a variety of qualitative properties of condensates were observed across studies and within studies (Fig. 1*A*iv). All groups observed at least some conditions where N-protein condensates exhibited apparent liquid-like behavior and/or morphology. However, a subset of studies also observed a number of conditions that favored the formation of solid-like/amorphous condensates. Specifically, solid-like condensates were dependent upon N-protein concentration (30), pH (32), RNA concentration (30, 31, 33, 35), type of RNA (7, 20, 21, 28, 30, 32, 35), type of salt (35), deletion of the SR domain (7), crowding-agent concentration (31, 35), M^104–222^-protein concentration (7), and incubation time prior to imaging (29). In another preliminary study (56), the N protein was reported to form both liquid-like and solid-like condensates capable of binding amyloid-specific dyes, which were modulated by the RNA:N protein ratio and driven by the IDR_central_ (including the SR domain). Purification conditions (particularly, contaminating nucleic acids that copurify with N protein) and sample preparation may also influence PS characteristics exhibited by the N protein (57). Collectively, these factors (combined with the previously noted association between SR domain phosphorylation and condensate characteristics) suggest that “phase separation” by the N protein can result in condensates with a wide range of material states and is highly sensitive to reaction conditions and PTMs.

In some sense, this collection of observations provides a more complete view of the suite of biophysical behaviors accessible to the N protein. However, they also highlight that caution should be exercised when extrapolating *in vitro* PS results to *in vivo* biophysical characteristics, since *in vivo* conditions certainly deviate from *in vitro* conditions, and since *in vivo* condensates typically involve many more molecular components than those included in simple PS experiments. Indeed, in studies that report assessment of condensate dynamics *in vivo* (typically by FRAP), N-protein foci unanimously exhibit liquid-like behavior (7, 20, 21, 25, 28, 29, 33, 34, 36), often with exchange rates faster than those observed for N-protein condensates *in vitro*.

## The SARS-CoV-2 N protein interacts with key stress granule nucleators, which may suppress or dissolve stress granules

PS is often associated with the formation of (or recruitment to) membraneless organelles, many of which also exhibit liquid-like behavior *in vivo* (10). Early studies characterizing physical interactions between SARS-CoV-2 proteins and human host proteins indicated an interaction between the N protein and key stress granule nucleating proteins, G3BP1 and G3BP2 (58, 59). Certain viruses can induce stress granule formation, co-opting stress granules to serve as viral replication or translation centers (17). Other viruses appear to suppress stress granule formation (17).

A number of follow-up studies supported the initial observation that the SARS-CoV-2 N protein physically interacts with G3BP1/2 (7, 27, 28, 31, 60). In three studies, the N protein either colocalized with stress granules (29, 33) or recruited G3BP1 to N-protein condensates in the absence of stress (7). Additionally, six other groups found that the N protein specifically interacted with G3BP1/2, leading to a reduction in the size and/or number of stress granules (26, 27, 31, 60, 61, 62, 63) (Fig. 1*B*i). *In vitro*, complete inhibition of G3BP1 PS could be achieved using only the IDR_NTD_ and NTD (residues 1–174). This inhibition was partially alleviated upon mutation of a short ITFG sequence (residues 15–18) in the IDR_NTD_ or three arginine residues (R92, R107, and R149) in the NTD (26), suggesting an important role for the ITFG motif and NTD arginine residues within the N protein in its interaction with G3BP1 and modulation of G3BP1 PS. Partial restoration of G3BP1 PS *in vitro via* mutation of these residues also correlated with partial restoration of stress granules *in vivo* (26, 63), and coexpression of competitive peptide inhibitors designed specifically to disrupt the ITFG-G3BP1 interaction reduced viral proliferation (63), indicating that the N-G3BP1 interaction is important for viral replication. Furthermore, one group observed an inverse correlation between the degree of N-protein co-PS with G3BP1 *in vitro* and the percentage of cells with stress granules *in vivo*, at least in the subset of constructs for which data were reported for both assays (31). This contributed to the interpretation that N-mediated stress granule inhibition was preceded by G3BP1/2 interaction, leading to initial recruitment of the N protein to stress granules prior to stress granule inhibition (31). Stress granule suppression could be modulated by asymmetric dimethylation of a single arginine residue (R95) in the N-protein RNA-binding domain (62). Furthermore, methylation of the same arginine, as well as an arginine within the SR domain (R177), promoted interaction with the 5′ UTR of SARS-CoV-2 gRNA and enhanced viral replication in mammalian cells (62), which may also regulate the formation of N+RNA condensates *in vivo* (21).

Collectively, these results suggest that (1) the effect of the N protein on stress granule inhibition varies based on one or more experimental conditions, and/or (2) the observed colocalization of N protein with stress granules is metastable and may eventually lead to stress granule inhibition or dissolution.

## N-protein PS is implicated in viral gRNA condensation and packaging

Nucleocapsid proteins are typically involved in viral genome packaging, and PS by nucleocapsid proteins of other viruses has been shown to mediate viral nucleic acid packaging [as discussed in (19)]. Thus, multiple studies have examined a role for N-protein PS in SARS-CoV-2 gRNA condensation and packaging (Fig. 1*B*ii) (19).

While many types of RNA (including nonviral RNA) are capable of inducing N-protein PS *in vitro* (Fig. 1*A*i), the potency of induction and properties of resulting condensates are influenced by the type of RNA and length of RNA used in co-PS experiments (Fig. 1*A*iv). Iserman *et al.* (21) observed that, even within the SARS-CoV-2 gRNA, different regions exerted different effects on N-protein PS, with the 5′ and 3′ ends promoting N-protein PS but more central regions promoting N-protein solubilization (*i.e.*, among the limited set of regions tested). The PS-promoting behavior of the 5′ and 3′ ends was also strand-specific, as complementary RNAs displayed solubilizing activities much like the central regions. Based on these observations, they proposed that the combination of PS-promoting and PS-solubilizing regions may help organize the SARS-CoV-2 RNA for efficient packaging and provide specificity for full-length gRNA over subgenomic RNA and host-cell RNA. Indeed, the presence of a small number of high-affinity N-protein binding sites specifically positioned near the termini of the SARS-CoV-2 gRNA was proposed to be critical to favor both association of gRNA ends (gRNA “cyclization”) and compaction of single RNA molecules over condensates containing multiple gRNA molecules (21, 64). However, in a separate study (30), N-protein PS was also induced by one of the central regions that exhibited a solubilizing effect in (21). The fragment tested in (30) was only 318 nt in length and started 67 nt upstream of the 581 nt long fragment tested in (21), so slight differences in sequence, length, or reaction conditions—particularly, protein and RNA concentrations, which also differed substantially between the two publications—might explain this discrepancy. Finally, the formation of condensates by N protein alone and in the presence of RNA was reportedly increased at biologically relevant temperatures (21), though two other studies observed little to no effect on condensate formation by N protein in the presence of RNA *in vitro* (28, 30).

Cubuk *et al.* (22) proposed a model in which a small number of packaging sequences within the gRNA act as high-affinity sites for N protein, creating local clusters of N protein. In this model, a kinetic energy barrier prevents the efficient fusion of N+RNA clusters, favoring single-molecule compaction of gRNA over the formation of larger, multi-RNA condensates. The authors note that, although the resulting behavior of N protein and gRNA would differ from classical PS at the microscopic level, similar types of intramolecular and intermolecular interactions may drive both processes, so observations of microscopic PS could serve as useful indicators of molecular-level behavior despite being an imperfect reflection of single-RNA compaction. However, this also may require further experimentation, since some examples of N+RNA co-PS do not directly correspond to RNA packaging *in vivo* [*e.g.*, polyA RNA robustly induces N-protein PS (32) and is present on both genomic and subgenomic RNA (65), yet polyadenylated subgenomic RNA is thought to be excluded from packaged coronavirus virions (66)]. Factors such as the length of these RNA features may be at least partially responsible for the apparent discrepancies.

While these models do not perfectly overlap, both groups suggest that a small number of gRNA sites with high-affinity N-protein binding sites could preferentially drive single-gRNA compaction and provide specificity for gRNA while excluding subgenomic and host-cell RNA (21, 22, 64).

## N-protein co-PS with M protein might anchor N+gRNA condensates to sites of virion budding at the ERGIC

SARS-CoV-2 is an enveloped virus: as such, compacted SARS-CoV-2 gRNA must be encapsulated in a protein-decorated membrane to form mature virions, which, in the case of SARS-CoV-2, occurs at the ERGIC membrane (3, 67). The SARS-CoV-2 M protein is a transmembrane protein that localizes to the ERGIC membrane to anchor and organize compacted gRNA at virion budding sites *via* interactions with other SARS-CoV-2 structural proteins (3, 67). The observed co-PS between the N protein and gRNA fragments, as well as co-PS between the N protein and the M^104–222^ protein (the soluble, cytosolic-facing fragment of the M protein), provided an enticing link between N+gRNA condensation and the localization of those condensates at the appropriate intracellular sites (Fig. 1*B*iii) (7). M^104–222^ co-PS was observed with N protein but not with RNA. Strikingly, three-component mixtures of N protein, gRNA fragments, and M^104–222^ resulted in the formation of nonuniform condensates comprised of an N+RNA “core”, decorated by a punctate M^104–222^ “shell” (7). Based on these results, the authors proposed a model whereby the N protein acts as a bridge between mutually exclusive N + M condensates and N+gRNA condensates. However, future experiments will be required to examine whether the full-length, membrane-bound M protein can undergo PS with the N protein; whether mutually exclusive condensates mediate this process *in vivo*; whether N+gRNA compaction/condensation occurs directly at M protein sites or is subsequently recruited after gRNA compaction; and how (mechanistically) the N protein might act as a bridge between condensates.

## N-protein PS antagonizes PS by the mammalian MAVS protein to suppress type I interferon production but promotes NF-κB signaling and cytokine production

In broad terms, cellular innate immune responses involve detection of foreign, pathogen-derived molecules that trigger a signaling cascade, culminating in activation of particular transcription factors such as NF-κB and IRF3 (68, 69). Activation of NF-κB and IRF3 upregulates expression of proinflammatory cytokines and type-I interferons, respectively. Double-stranded viral RNA is a common pathogen-derived molecule that can be sensed by mammalian proteins such as RIG-I and MDA5, which typically initiates IRF3 activation and expression of type-I interferons (IFNs), particularly IFN-β (70). One intermediate in this cascade, MAVS, forms prion-like aggregates upon activation by RIG-I, which in turn leads to activation of IRF3 and subsequent expression of IFNs (71, 72). However, a number of viruses manipulate host-cell innate immune responses specifically linked to stress granule formation resulting from viral infection (17, 19).

In a recent study, the N protein was found to inhibit the production of IFN-β and coimmunoprecipitate with MAVS (25), leading to the hypothesis that N protein might interfere with MAVS self-association (Fig. 1*B*iv). Prion-like activity is distinct from, but often associated with, PS behavior. Indeed, the MAVS protein was capable of undergoing PS in the absence of N protein. However, in stark contrast to the N-protein co-PS examples above, where partner molecules tended to enhance N-protein PS, MAVS PS was anticorrelated with N-protein PS. Furthermore, deletion of the N-protein CTD fully restored MAVS PS and IFN-β expression, while mutation or acetylation of a key lysine residue within the N protein, K375, could partially restore MAVS PS and IFN-β expression. Based on these observations, the authors proposed that the PS propensity of the N protein directly correlates with suppression of type-I IFNs. Indeed, a separate group found that a common N-protein polymorphism, R203K/G204R, enhanced both N-protein PS and N-protein-mediated suppression of IFN expression (20), which is consistent with the model of MAVS PS suppression and concomitant inhibition of IFN-β expression by N-protein PS. However, it is also important to note that other SARS-CoV-2 proteins are capable of suppressing the type-I IFN response by distinct mechanisms (73, 74, 75, 76, 77, 78, 79, 80, 81), so inhibition of MAVS signaling by the N protein is not solely responsible for innate immune suppression during SARS-CoV-2 infection.

While the IRF3/IFN-β branch of innate immunity is suppressed by SARS-CoV-2 N-protein PS, NF-κB signaling is activated by N-protein PS *via* recruitment of the kinases TAK1 and IKKβ to N-protein condensates (Fig. 1*B*iv) (34). N-protein-mediated activation of NF-κB signaling corresponded to an increase in the production of proinflammatory cytokines. Deletion of the N-protein CTD or treatment with 1,6-hexanediol disrupted N-protein PS and dampened the NF-κB-mediated induction of cytokines. Given that overproduction of cytokines and weak type-I IFN induction are defining features of COVID-19 (82), NF-κB activation by N-protein PS represents at least one plausible mechanism by which this might occur. While this study also reported upregulation of IFN-β upon SARS-CoV-2 infection *in vitro*, this occurred in a delayed manner, was not explored in greater detail, and was not explicitly linked to N-protein PS (34). Therefore, SARS-CoV-2 differentially regulates distinct branches of innate immune pathways, which may contribute to the pathological features unique to SARS-CoV-2.

Collectively, these results suggest that interaction between MAVS and the SARS-CoV-2 N protein competes for the interaction sites necessary for both MAVS PS and N-protein PS, and this competition may interfere with the production of key IFNs in response to SARS-CoV-2 infection *in vivo*. Conversely, N-protein PS activates the NF-κB signaling pathway, resulting in overactivation of cytokine production, especially in severe SARS-CoV-2 cases. Multiple groups have proposed a two-stage progression of SARS-CoV-2 pathology involving early suppression of innate immune pathways, followed by hyperactivation (73, 83, 84, 85, 86). Therefore, additional studies may be required to temporally and mechanistically resolve the influence exerted by SARS-CoV-2 on innate immune pathways throughout the course of infection.

## N-protein condensates recruit viral RNA-dependent RNA polymerase, which may promote viral genome replication

Biomolecular condensates are complex mixtures of particular proteins and RNAs enriched to varying degrees (10, 87). For some condensates, this can have a catalytic effect, accelerating certain reactions when the necessary components are concentrated within the condensates (10). A number of viruses induce the formation of viral “replication centers” (also known as “replication factories” or “replication compartments”) enriched in the proteins and nucleic acids necessary for viral genome replication in host cells. At least a subset of these replication centers are membraneless organelles with liquid-like properties consistent with phase-separated condensates (17, 18).

In two studies, condensates formed by the SARS-CoV-2 N protein and RNA recruited components of the SARS-CoV-2 RNA-dependent RNA polymerase (RdRp) complex responsible for replicating viral gRNA (29, 36). The degree of recruitment of RdRp components to condensates decreased upon phosphorylation of the SR domain of the N protein (29), consistent with the increased dynamics and reduced PS of phosphorylated N protein also observed in that study. While these results provide important preliminary support for a connection between N-protein PS and the formation of SARS-CoV-2 replication centers (Fig. 1*B*v), additional experiments will be required to demonstrate that RdRp is recruited to N-protein condensates *in vivo*, and that RdRp recruitment actually enhances viral RNA replication. Furthermore, if N protein and viral RNA co-PS mediate both viral RNA packaging and viral RNA replication, it is unclear whether these processes are mutually exclusive. If so, SARS-CoV-2 would presumably possess mechanisms to spatiotemporally regulate these essential steps *in vivo*.

## Conclusions

Although *in vitro* PS experiments appear simple at first glance, these assays can be highly sensitive to purification methods and buffer conditions (88). A series of general “best practices” have been proposed for PS experiments, yet optimal strategies must also be specifically tailored for certain proteins (88). Despite the relatively few experimental parameters typically involved in PS experiments, experimenter choice with respect to each parameter can vary greatly. Therefore, independent labs examining PS of the same protein would be unlikely to perform perfectly identical experiments from a procedural standpoint. Parallel work from multiple labs can result in iterative experimentation with slight variation, potentially revealing observations that are robust across a number of experimental approaches, conditions, and experimenters. However, our current scientific system—which disproportionately rewards and incentivizes novelty over reproducibility—results in missed opportunities if work that overlaps heavily with previously published work becomes more difficult to publish, is abandoned entirely, or is never attempted to begin with.

These individual incentives were superseded by the collective importance of combatting SARS-CoV-2, leading many independent labs to perform and publish strongly overlapping sets of experimental results. Far from being redundant, this collection of results has provided a more complete map of biophysical behavior exhibited by the SARS-CoV-2 N protein across a range of conditions. *In vitro*, N-protein PS was consistently regulated by factors commonly influencing electrostatic interactions in the formation of condensates, including salt concentration, pH, RNA concentration, type of RNA, and phosphorylation within the SR domain. Many of these factors affected not only the degree of condensate formation but also the material states of condensates, favoring either liquid-like or solid-like condensates. Additionally, while any pair of individual studies might emphasize different regions of the N protein as important for PS, a consensus view across the full suite of studies helps illuminate which N-protein domains are consistently involved in N-protein co-PS with RNA. Furthermore, a limited subset of these studies suggests that the domains important for N-protein PS were heavily dependent upon the co-PS partner molecule (RNA, G3BP1, M^104–222^ protein, or MAVS). This is particularly intriguing if the N-protein domain dependence on co-PS partner extends to *in vivo* behavior: not only might the N protein form compositionally distinct condensates with a surprisingly versatile set of proteins, but these condensates may also be independently regulated *via* modulation of different N-protein domains or distinct N-protein isoforms generated by proteolysis. However, the relationship between *in vitro* observations of N-protein PS and the formation of foci *in vivo* is not always clear. For example, an N-protein variant consisting of only the IDR_NTD_ and NTD strongly inhibited co-PS with G3BP1+RNA (Fig. 4*B*) and completely abolished co-PS with RNA (Fig. 2) yet strongly enhanced its ability to form foci *in vivo* in the absence of stress (Fig. 4*C*). Partner-dependent effects on the N-protein domains important for co-PS offer a plausible explanation for the observed differences between contexts, but this hypothesis is currently speculative, and more experiments are required to obtain complete coverage of N-protein deletions in different contexts.

Finally, the sequential publication model conceivably could affect the number of distinct functions discovered for a particular protein if initial reports of protein function lead to less experimental exploration for additional functions, which is especially relevant for multifunctional proteins (89). The diversity of studies on N-protein PS has linked this behavior to a remarkable array of possible *in vivo* functions, which may have been facilitated by parallel exploration. Functions proposed for N-protein PS include: the modulation of host-cell condensates, particularly stress granules; SARS-CoV-2 gRNA cyclization and compaction; the anchoring and packaging of N+gRNA complexes/condensates, in conjunction with the M protein, into nascent virions at the ERGIC membrane; the formation of viral replication centers; and the modulation of host-cell innate immune responses.

## Conflict of interest

The authors declare that there is no conflict of interest with the contents of this article.
